# Research on the Frequency-Dependent Halfwave Voltage of a Multifunction Integrated Optical Chip in an Interferometric Fiber Optic Gyroscope

**DOI:** 10.3390/s19132851

**Published:** 2019-06-27

**Authors:** Ran Bi, Lijun Miao, Tengchao Huang, Guangyao Ying, Shuangliang Che, Xiaowu Shu

**Affiliations:** 1 State Key Laboratory of Modern Optical Instrumentation, Zhejiang University, No. 38 Zheda Road, Hangzhou 310027, China; 2 State Grid Zhejiang Electric Power Research Institute, Hangzhou 310014, China

**Keywords:** interferometric fiber optic gyroscope, multifunction integrated optical chip, halfwave voltage, frequency dependence

## Abstract

The multifunction integrated optical chip (MIOC) is one of the most critical parts of the interferometric fiber optic gyroscope (IFOG), and research on the halfwave voltage of the MIOC is meaningful for a high-precision IFOG. In this paper, the correlation between the frequency and halfwave voltage, which affects the interference light intensity of IFOG, is presented theoretically. A widespread measurement method for frequency dependence of the halfwave voltage, based on lock-in amplification and sinusoidal modulation, is proposed. Further, the measurement result and the oscillation of interference light intensity in the Sagnac interferometer are presented, which are in great agreement with the theory. This paper proposes the frequency dependence of the halfwave voltage and provides a new error research direction for the improvement of the MIOC in a high-precision IFOG.

## 1. Introduction

The interferometric fiber optic gyroscope (IFOG) is a rotation sensor based on the optical common path interferometer, which is widely applied in navigation and positioning [1,2]. According to application requirements, such as a long-haul time, high precision is one of the main directions for the development of the IFOG in the future.

The multifunction integrated optical chip (MIOC) is the crucial component in the IFOG, which acts as a beam split/combination, a light polarizer, and a reciprocal phase modulator [1]. The optical polarization error and phase error of the MIOC place a non-ignorable restriction on the precision of the IFOG. Thereby, the study of the MIOC could lead to significant improvement in the IFOG accuracy characteristics.

Common characteristics of the MIOC, such as insertion loss [3], the polarization extinction ratio [4], residual intensity modulation [5], etc., have been studied in detail. However, research on the MIOC response error is still preliminary, such as phase drift [6], center wavelength stability [7], etc. Considering the voltage-phase conversion, some reports have focused on the halfwave voltage, especially a lower working voltage [8,9], temperature fluctuation [10,11,12], and wavelength correlation [12]. For example, Ran Ding et al. reported a sub-1 V halfwave voltage by a better radio frequency design and more active polymer doping [8]. Xianhui Mao et al. presented a halfwave voltage reduction when the temperature was increased [10]. Additionally, Fuling Yang et al. described the positive correlation between the halfwave voltage and wavelength [12]. However, as far as we know, there are no published works focused on the frequency dependence of halfwave voltage, due to nonlinear effects and structural resonance.

Herein, the frequency-dependent halfwave voltage and its specific effect on the IFOG is studied. Section 2 analyzes the origin of frequency dependence of the halfwave voltage and the oscillation of interference light intensity theoretically. In Section 3, a measurement method for frequency dependence of the halfwave voltage is proposed and the measurement result shows a great agreement with the theory. Section 4 is the discussion on the experimental results. Section 5 presents the conclusion, followed by the acknowledgments.

## 2. Theory and Simulation

### 2.1. Origin

The voltage-phase conversion of the MIOC is based on the electro-optical (EO) effects of LiNbO_3_, of which the Pockels effect is the most widely studied. However, in the high-precision IFOG, the additional EO effects will cause the distortion of electro-optic conversion.

Herein, the EO effect in the MIOC is considered as a combination of the following three effects: (1) The intrinsic EO effect, (2) the change of light path due to piezoelectric displacement, and (3) the photo-elastic effect due to the piezoelectric strain. Further, the optical retardation caused by the effects above can be defined as [13,14]

(1) $$\begin{array}{l} R_{1}=\frac{\pi n_{e}^{3}\gamma_{c}El}{\lambda }\\ R_{2}=\frac{2\pi \Delta n}{\lambda }\Delta l\\ R_{3}=\frac{\pi n_{e}^{3}l}{\lambda }\sum_{i,j,k,l}p_{i,j,k,l}S_{k,l} \end{array}$$

where *n_e_* is the extraordinary refractive index; *γ_c_* is the intrinsic EO coefficient; *E* is the E-field intensity; *l* is the light pass length; *λ* is the wavelength; Δ*n* is the initial birefringence independent of the E-field; Δ*l* is the change of light path length; and *p_i_*_,*j*,*k*,*l*_ and *S_k_*_,*l*_ are the photo-elastic coefficient and elastic strain, respectively.

Considering these effects, the equivalent EO coefficient can be expressed as [13,14]

(2) $$\gamma_{c}^{II}=\frac{\lambda }{\pi n_{e}^{3}El}\left[\frac{\pi n_{e}^{3}\gamma_{c}}{\lambda }El+\frac{2\pi \Delta n}{\lambda }\Delta l(f)+\frac{\pi n_{e}^{3}l}{\lambda }\sum_{i,j,k,l}p_{i,j,k,l}S_{k,l}(f)\right],$$

where Δ*l* and *S_k_*_,*l*_ are considered a function of frequency.

In order to better match the reality, a phase-lag between the E-field and piezoelectric displacement is introduced, and the overall EO coefficient is represented by [13,14]

(3) $$\gamma_{c}^{III}=\frac{\lambda }{\pi n_{e}^{3}El}\sqrt{{\left[R_{1}+\left(R_{2}+R_{3}\right)\cos\delta \right]}^{2}+{\left[\left(R_{2}+R_{3}\right)\sin\delta \right]}^{2}},$$

where *δ* is the phase-lag.

**Figure 1:**
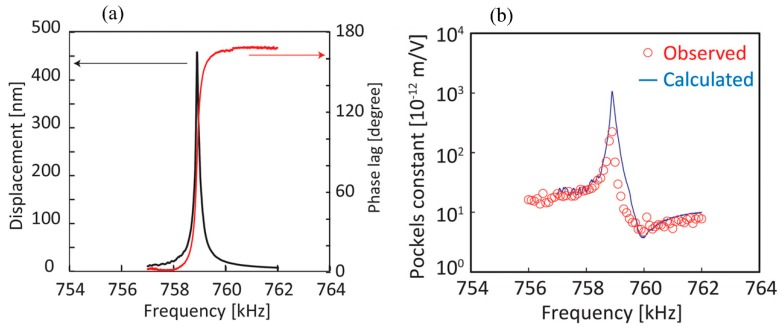
(**a**) Piezoelectric displacement and phase-lag between the E-field and piezoelectric displacement as a function of frequency; (**b**) calculated and observed electro-optical (EO)-coefficients as a function of frequency. Reprinted from [13], with the permission of AIP Publishing.

In Reference [13], the piezoelectric displacement and its phase-lag to the E-field were measured, as illustrated in Figure 1a. Figure 1b shows the EO coefficient calculated by Equation (3) and the experimentally observed data, which presents the consistency between the simulation and measurement [13].

In terms of the MIOC, the halfwave voltage, and the key parameter during voltage-phase converting, can be expressed as [15]

(4) $$V_{\pi }=\frac{G\lambda }{2\pi n_{e}^{3}\gamma_{c}^{III}\Gamma L},$$

where $\gamma_{c}^{III}$ is the overall EO coefficient, *G* is the one-arm two-electrode spacing in the MIOC, and Γ is the overlap factor between the electric field and the light field, *L* is the length of modulation electrode.

Further, the frequency dependence of $\gamma_{c}^{III}$, *G*, and *L* results in the differences of the halfwave voltage at different frequencies. The halfwave voltage frequency dependence can be represented by

(5) $$V_{\pi }(\omega )=V_{\pi s}[1-\sigma (\omega )],$$

where *V_πs_* is the reference halfwave voltage, *σ*(*ω*) is the deviation ratio at different frequencies and can be expressed as

(6) $$\sigma (\omega )=1-\frac{G(\omega )/G_{0}}{\gamma_{c}^{III}(\omega )/\gamma_{c0}^{III}*L(\omega )/L_{0}},$$

where $\gamma_{c}^{III}\left(\omega \right)$, *G*(*ω*), and *L*(*ω*) are the overall EO coefficient, one-arm two electrode spacing, and the length of modulation electrode at frequency ω; $\gamma_{c0}^{III}$, *G*_0_, and *L*_0_ are the corresponding reference value. 

Reference [13] is based on the LiNbO_3_ crystal, and the results in Figure 1a were measured by a laser doppler vibrometer connected to a lock-in amplifier. However, the actual MIOC structure is intricate and has many constraints, and the halfwave voltage frequency dependence is too complicated to be measured from the structural deformation. The measurement in Reference [13] cannot be used for MIOC devices. Thereby, a feasible method for the frequency dependence of the MIOC halfwave voltage, especially MIOC devices, hasn’t been proposed.

### 2.2. Effect on Interference Light Intensity

In the IFOG, the square wave modulation is one of the most commonly used modulation schemes, and the modulation waveform can be expressed as [1]

(7) $$U_{b}\left(t\right)=\left\{ \begin{array}{l} \begin{array}{cc} \frac{U_{0}}{2} & 0\leq t<\tau \end{array}\\ \begin{array}{cc} -\frac{U_{0}}{2} & \tau \leq t<2\tau \end{array} \end{array}\right.,$$

where *τ* is the transit time when the light wave propagates along the fiber ring, and *U*_0_ is the amplitude of the modulation wave.

After the Fourier transform, the square wave can be represented by a superposition of multiple sinusoidal signals whose frequencies are odd multiples of the fundamental frequency, which can be expressed as

(8) $$U_{b}(t)=\frac{2U_{0}}{\pi }\sum_{i=1}^{\infty }\frac{1}{2i-1}\sin[(2i-1)\omega_{0}t],$$

where *i* is a positive integer.

Due to the time delay difference modulation and interference response principle in the IFOG, the interference intensity can be expressed as

(9) $$\begin{array}{l} I=I_{0}\left\{1+\cos\left[\Delta \phi_{R}+2U_{b}(t)*\frac{\pi }{V_{\pi }(\omega )}\right]\right\}\\ =I_{0}\left\{1+\cos\left\{\Delta \phi_{R}+4U_{0}\sum_{i=1}^{\infty }\frac{\sin[(2i-1)\omega_{0}t]}{\left(2i-1)*V_{\pi }[(2i-1)\omega_{0}\right]}\right\}\right\}\\ =I_{0}\left\{1+\cos\left\{\Delta \phi_{R}+\phi_{B}-4U_{0}\sum_{i=1}^{\infty }\frac{\sin[(2i-1)\omega_{0}t]}{2i-1}\frac{\sigma [(2i-1)\omega_{0}]}{V_{\pi }[(2i-1)\omega_{0}]}\right\}\right\}\\ \approx I_{0}+I_{0}\cos\left(\Delta \phi_{R}+\phi_{B}\right)-I_{0}\sin\left(\Delta \phi_{R}+\phi_{B}\right)\sum_{i=1}^{\infty }\frac{4\sigma [(2i-1)\omega_{0}]U_{0}}{(2i-1)V_{\pi s}}\sin\left[(2i-1)\omega_{0}t\right] \end{array}$$

where *V_π_*(*ω*) and *σ*(*ω*) are the halfwave voltage and its deviation ratio when the modulation frequency is *ω*, respectively; *I*_0_ is the light source intensity; Δ*ϕ_R_* is the Sagnac phase; and *ϕ_B_* is the ideal modulation phase, which is

(10) $$\phi_{B}=\pm \frac{\pi U_{0}}{V_{\pi s}}.$$

The first two terms of Equation (9) are a theoretical interference expression when *V_π_* is not a function of frequency, and the remaining one is the “oscillation” in the interference light intensity, whose frequencies are the odd multiple of the modulation fundamental frequency. Considering the linear superposition in Equation (9), the total oscillation is the sum of oscillation at different frequencies. By randomly selecting one frequency term, the interference oscillation can be presented as

(11) $$I_{osc,m}=I_{0}\sin\left(\Delta \phi_{R}+\phi_{B}\right)\frac{4\sigma [(2m-1)\omega_{0}]U_{0}}{(2m-1)V_{\pi s}}\sin\left[(2m-1)\omega_{0}t\right],$$

where (2*m* − 1)*ω*_0_ is the selected oscillation frequency and *I_osc,m_* is the oscillation at (2*m* − 1)*ω*_0_ frequency.

When considering one frequency, from Equation (11), the oscillation amplitude is related to the halfwave voltage deviation ratio and modulation voltage amplitude. For a MIOC device, the frequency dependence of the halfwave voltage is fixed in a stable environment, which means *σ*(*ω*) is fixed. Thereby, the peak-to-peak value of interference oscillation is a function of modulation voltage, which can be expressed as

(12) $$I_{pp}=\left|I_{0}\frac{8\sigma [(2m-1)\omega_{0}]U_{0}}{(2m-1)V_{\pi s}}\sin\left(\Delta \phi_{R}+\frac{U_{0}\pi }{V_{\pi s}}\right)\right|.$$

Considering the linear superposition in Equation (9), when the modulation amplitude is an integer multiple of *V_πs_*, the total interference oscillation is eliminated, but the sensitivity of the IFOG is also suppressed [1].

## 3. Experiment

### 3.1. Measurement Method

**Figure 2:**
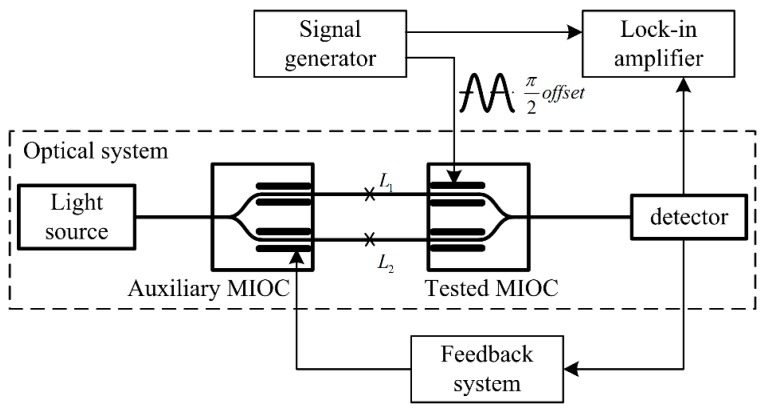
Schematic of the measurement method for frequency dependence of the multifunction integrated optical chip (MIOC) halfwave voltage.

For accurate measurement of the MIOC halfwave voltage [16] at different frequencies, it is necessary to adopt a single frequency modulation wave. Based on the sinusoidal modulation scheme, the measurement method is shown in Figure 2, where the feedback system is used to suppress the slow phase shift introduced by the influence of environment and stress. The feedback system tests the average phase of the interference intensity. Additionally, when the average phase isn’t π/2, the feedback system will adjust the voltage on the auxiliary MIOC to make the average phase back to π/2.

In Figure 2, it is not necessary to apply a common path interference structure. The auxiliary MIOC, whose splitting ratio is approximately 50:50, acts as a splitter. Moreover, the auxiliary MIOC and tested MIOC are interchangeable, and the pigtails of MIOCs are spliced at a zero angle. Furthermore, considering the short coherence length of a wide-spectrum light source, a laser light source is used in Figure 2.

A sinusoidal modulation signal, whose frequency is *ω* and amplitude is *U*_0_, is applied to the tested MIOC by the signal generator, and an offset modulation signal, which is half of the halfwave voltage, is applied on the auxiliary MIOC. Referring to the relevant theory of sinusoidal modulation, the interference light intensity can be expressed as

(13) $$\begin{array}{ll} I & =I_{0}\left\{1+\cos\left[\frac{U_{0}\pi }{V_{\pi }(\omega )}\sin(\omega t)+\frac{\pi }{2}\right]\right\}\\ & =I_{0}\left\{1-\sin\left[\frac{U_{0}\pi }{V_{\pi }(\omega )}\sin(\omega t)\right]\right\}\\ & =I_{0}-2I_{0}\sum_{i=1}^{\infty }J_{2i-1}\left(\frac{U_{0}\pi }{V_{\pi }(\omega )}\right)\cos[(2i-1)\omega t] \end{array}$$

where, *I*_0_ is the light source intensity, *V_π_*(*ω*) is the halfwave voltage as a function of frequency, and *J_n_* is the *n*-order Bessel function.

Using a lock-in amplifier to detect its first harmonic, the output can be obtained as

(14) $$I_{f}=-2I_{0}J_{1}\left(\frac{U_{0}\pi }{V_{\pi }(\omega )}\right).$$

Based on Equation (14) and the lock-in amplifier output, the halfwave voltage at a modulation frequency can be calculated.

In this method, fluctuations of the external environment will cause instability of the lock-in amplifier output. Thereby, for optimization, a voltage feedback system is applied for suppressing the fluctuations. In addition, the lengths of fibers, *L*_1_ and *L*_2_, in Figure 2, need to be approximately the same. Moreover, the splitting ratios of the MIOCs are approximately 50:50. When considering the deviation of the splitting ratio, Equation (14) can be written as

(15) $$I_{f}=-4\sqrt{\frac{\xi_{1}\xi_{2}}{{\left(\xi_{1}+\xi_{2}\right)}^{2}}}I_{0}J_{1}\left(\frac{U_{0}\pi }{V_{\pi }(\omega )}\right),$$

where *ξ*_1_:*ξ*_2_ is the splitting ratio of MIOC.

By testing the extremum of the interference light intensity, the effect of splitting ratio on the measurement results can be eliminated.

### 3.2. Experiment Result

**Figure 3:**
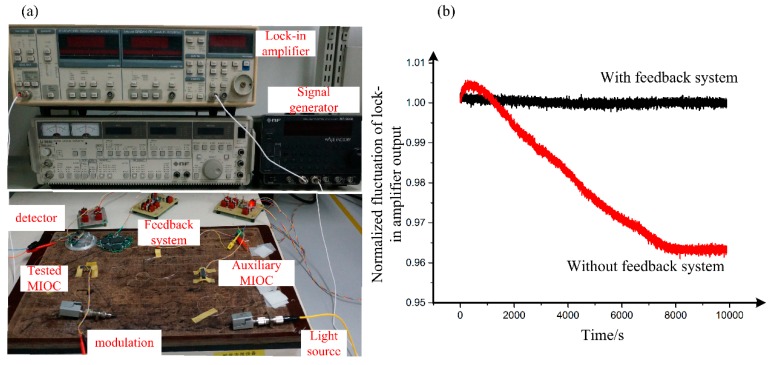
(**a**) Measurement setup for the halfwave voltage as a function of frequency; (**b**) normalized output of lock-in amplifier with and without the feedback system.

**Figure 4:**
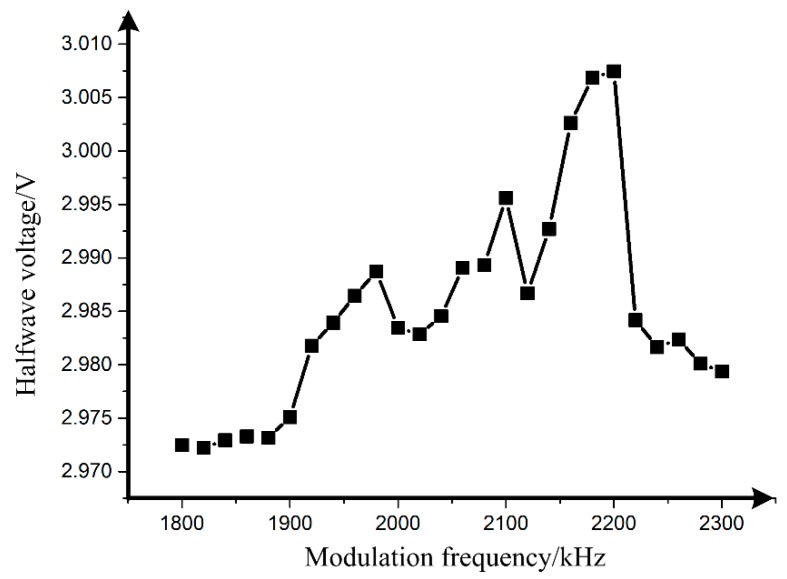
Measurement result of the frequency-dependent halfwave voltage.

The actual measurement system for the frequency dependence of the MIOC halfwave voltage is illustrated in Figure 3a, while the suppression effect of the feedback system on the output fluctuation of lock-in amplifier is in Figure 3b. Additionally the halfwave voltage of different frequencies is shown in Figure 4.

In the measurement, the amplitude of the modulation sinusoidal wave is fixed at 1 V, and the scanning frequency range is 1.8 MHz to 2.3 MHz. As can be seen from Figure 4, the halfwave voltage of the MIOC is about 3.007 V when the modulation frequency ranges from 2.18 MHz to 2.2 MHz, while it is about 2.973 V at 1.9 MHz and below. Further calculation indicates that the halfwave voltage deviation ratio at 2.18 MHz is 1.14%. The measurement result verifies the existence of the frequency-dependent halfwave voltage.

**Figure 5:**
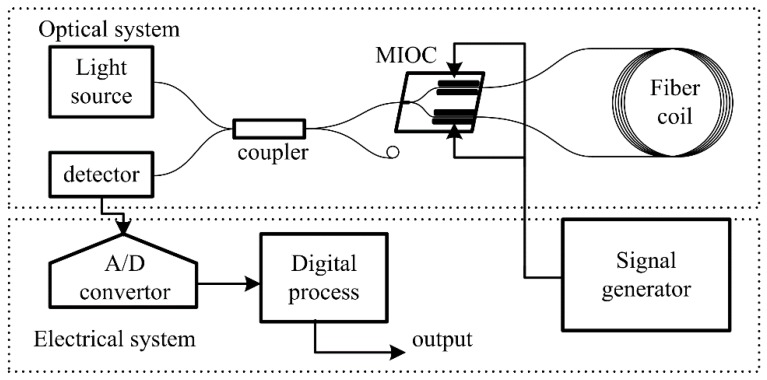
Schematic of the Sagnac interferometer.

**Figure 6:**
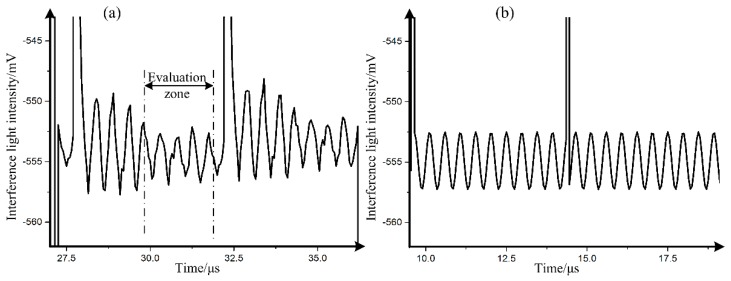
(**a**) Interference light intensity signal obtained by the A/D convertor; (**b**) interference light intensity signal by simulation with the measurement results.

In order to study the effect of the frequency-dependent halfwave voltage on the IFOG, the Sagnac interferometer, whose parameters are shown in the Table 1, is built using the MIOC device, as illustrated in Figure 5. From Table 1, it can be seen that if there is no modulation on MIOC, then the output after the detector is 2.01 V. In Figure 5, the eigen frequency modulating square wave with a 1.5 V amplitude is applied on the MIOC. Then, the interference light intensity signal after the detector, whose time average (40 ms) is shown in Figure 6a, is obtained by the A/D converter, whose resolution is 1.22 mV and sample rate is 20 MHz. A 2.18 MHz oscillation dominates the interference light intensity, which is 21 times the modulation frequency. Considering the parameters in Table 1 and the measured results above, the simulation of interference light intensity by Equation (11) is shown in Figure 6b. The difference between Figure 6a,b is caused by the distortion of the interference [6].

**Figure 7:**
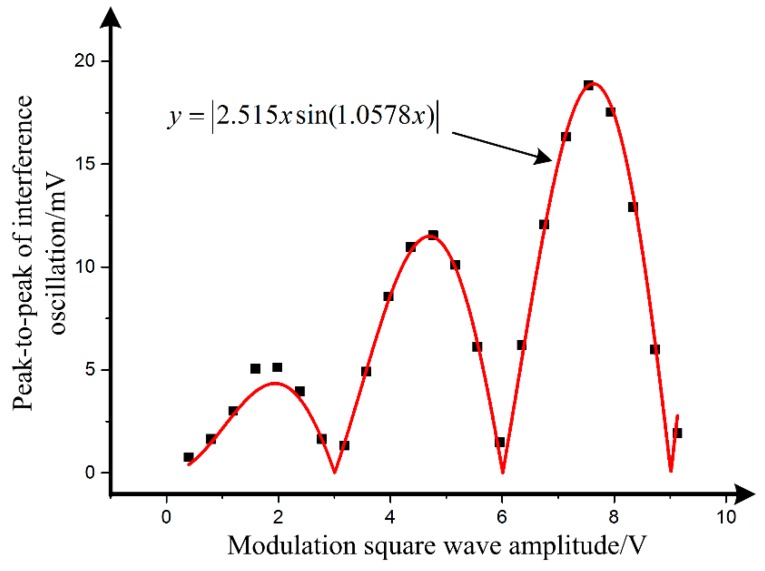
Measured peak-to-peak value of interference oscillation as a function of modulation square wave amplitude and its model fitting.

By selecting the second half of the stable segment of the curve as the evaluation area, a 5 mV peak-to-peak value of interference oscillation is calculated. Subsequently, by changing the amplitude of the modulation square wave, the measured relationship between the peak-to-peak value of interference oscillation and modulation amplitude is shown in Figure 7. The black points are the measurement results when the modulation amplitude changes, and the red line in Figure 7 is the model fitting using Equation (12).

The result of model fitting can be written as

(16) $$\begin{array}{c} V_{\pi }=2.97\;V\\ \sigma (2.18\;\mathrm{MHz})=0.98\% \end{array},$$

which is in good agreement with the theory in Section 2 and the experimental result, 1.14%, in Figure 4.

## 4. Discussion

**Figure 8:**
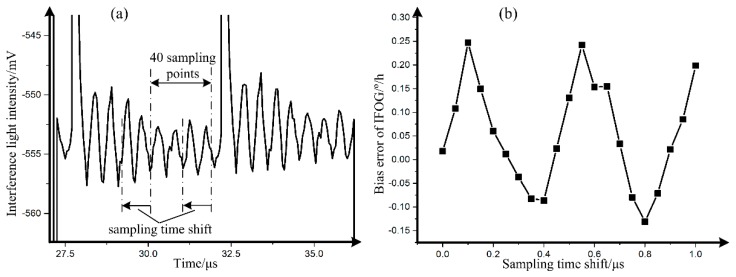
(**a**) Schematic diagram of sampling points; (**b**) measured results between the bias error of IFOG and sampling time shift.

The performance of the IFOG is directly limited by the interference light intensity signal [1]. In Reference [14], the effect of interference oscillation on the performance of IFOG was presented. Herein, based on the IFOG system in Section 3, the 40 sampling points average is adopted, as shown in Figure 8a. When the sampling time is shifted, the bias error, also called an angular velocity error of the IFOG, is shown in Figure 8b, which was tested in the Earth’s rotation rate (7.56 °/h at laboratory location). The angular random walk and bias drift, calculated by the Allan deviation [1], were unchanged with the sampling time shift.

The most fundamental solution is the sinusoidal modulation scheme, but there are other issues that need to be addressed [1], which needs further studies. Moreover, after sampling the interference light intensity in IFOG, a digital notch filter, whose stop frequency is the same with the interference oscillation, can be adopted for suppression.

Compared with Reference [14], a new performance for the MIOC resonant effect, the frequency dependence of the halfwave voltage, is presented, which is more comprehensive for the evaluation of MIOC. This paper is focused on LiNbO_3_ based MIOCs, but the study, the theory and the measurement method are suitable for all types of MIOCs, which might have more than one harmonic frequency. If the measurement method is used for another MIOC, the frequency dependence of the halfwave voltage might be another, due to the different materials and structures.

## 5. Conclusions

This article focuses on the frequency dependence of the MIOC halfwave voltage in the IFOG. The significant effect of the frequency-dependent halfwave voltage on the interference light intensity, which affects the performance of the IFOG, is presented theoretically. Subsequently, a method based on lock-in amplification is found to measure the halfwave voltage of the MIOC at different frequencies. The measurement result shows that the peak halfwave voltage deviation ratio at resonance frequency is 1.14%. Finally, based on the Sagnac interferometer, the interference oscillation is demonstrated. This research proposes new requirements for the MIOC, and provides a new idea for the development of a high-precision IFOG.

## Figures and Tables

**Table 1:** Parameters of interferometric fiber optic gyroscope (IFOG).

Parameter	Value
Source optical power	470 μW
Total optical losses	18 dB
Detector responsivity	270 V/mW
Radius of fiber coil	0.1 m
Transit time	4.8 μs
Eigen frequency	104.1 kHz
Detector output when no light input	−2.533 V
